# [Retracted] miR-760 mediates hypoxia-induced proliferation and apoptosis of human pulmonary artery smooth muscle cells via targeting TLR4

**DOI:** 10.3892/ijmm.2025.5642

**Published:** 2025-09-22

**Authors:** Yu-Zhong Yang, Yun-Feng Zhang, Lei Yang, Jing Xu, Xu-Ming Mo, Wei Peng

Int J Mol Med 42: 2437-2446, 2018; DOI: 10.3892/ijmm.2018.3862

Following the publication of this paper, it was drawn to the Editor's attention by a concerned reader that, in addition to the duplication of data comparing Fig. 4B with Fig. 6F (showing the results of Transwell assay experiments) and within Fig. 6C (showing the results of scratch-wound assay experiments), a data panel in Fig. 2C was also featured in a paper written by different authors that had already been submitted to the journal *Cancer Science*.

Owing to the fact that the contentious data in the above article were found to be strikingly similar to data that had already been submitted for publication elsewhere, the Editor of *International Journal of Molecular Medicine* has decided that this paper should be retracted from the Journal. The authors were asked for an explanation to account for these concerns, but the Editorial Office did not receive a satisfactory reply. The Editor apologizes to the readership for any inconvenience caused.

